# A new family with hereditary lysozyme amyloidosis with gastritis and inflammatory bowel disease as prevailing symptoms

**DOI:** 10.1186/1471-230X-14-159

**Published:** 2014-09-13

**Authors:** Estelle Jean, Mikael Ebbo, Sophie Valleix, Lucas Benarous, Laurent Heyries, Aurélie Grados, Emmanuelle Bernit, Gilles Grateau, Thomas Papo, Brigitte Granel, Laurent, Daniel, Jean-Robert Harlé, Nicolas Schleinitz

**Affiliations:** Departement of Internal Medicine, APHM, Aix-Marseille Université, Marseille, France; Departement of Gastroenterology, APHM, Marseille, France; Department of Internal Medicine, APHP, Hôpital Tenon, Paris, France; Department of Internal Medicine, APHP, Centre Hospitalier Bichat, Paris, France; Departement of Pathology, APHM, Aix-Marseille Université, Marseille, France

**Keywords:** Amyloidosis, Lysozyme, Gastritis, Rectocolitis

## Abstract

**Background:**

Systemic amyloidoses is a heterogeneous group of diseases either acquired or hereditary. Amyloidoses can involve the gastrointestinal tract and the nature of the precursor protein that forms the fibrils deposits should be identified to adjust the treatment and evaluate the prognosis. Lysozyme amyloidosis (ALys) is a rare, systemic non neuropathic hereditary amyloidosis with a heterogenous phenotype including gastrointestinal, renal and hepatic symptoms.

**Case presentation:**

We report and describe symptoms and gastrointestinal tract involvement in a new family with hereditary lysozyme amyloidosis. Clinical manifestations and organ involvement of nine affected members of a new family with the p.Trp82Arg ALys variant were recorded. All affected individuals suffered with prevailing gastrointestinal symptoms leading to the diagnosis of ALys. 8/9 had non specific upper gastrointestinal symptoms and 3/9 had rectocolic inflammation evoking inflammatory bowel disease. No other organ involvement by amyloidosis was found. Histological examination revealed amyloid deposits in all cases and all carried the p.Trp82Arg ALys variant at a heterozygous state.

**Conclusion:**

Hereditary amyloidosis associated with the p.Trp82Arg lysozyme variant in this new family is predominantly associated with mild upper gastrointestinal tract involvement and in some cases with inflammatory bowel disease. Amyloidosis should be considered in atypical or treatment resistant, upper or lower chronic gastrointestinal symptoms. When associated with a familial history a lysozyme gene mutation must be searched.

## Background

Systemic amyloidoses is characterized by deposition of protein in an abnormal fibrillary conformation leading to disruption of tissues and potential organ dysfunction. Systemic amyloidoses can be either acquired or hereditary. Acquired amyloidosis, mainly related to AA and AL amyloidosis, should be distinguished from hereditary neuropathic and non-neuropathic systemic amyloidosis. Lysozyme amyloidosis (ALys) belongs to the group of hereditary non-neuropathic systemic amyloidoses, firstly described in 1932 by Ostertag [1] with the identification of the lysozyme variant performed in 1993 by Pepys et al. [2] (Online Mendelian Inheritance in Man 153450). Until now eight pathogenic mutations of the lysozyme gene (*LYZ*) have been reported [2–8]. All are inherited in an autosomal dominant fashion. ALys phenotype is heterogeneous and includes gastrointestinal (GI) symptoms, hepatic rupture, sicca syndrome, purpura, renal failure and lymphadenopathy. We report here a new large family in which the p.Trp82Arg variant (this nomenclature includes the 18 amino acid signal peptide according to Human Genome Variation Society guidelines, http://www.hgvs.org/mutnom// and p.Trp82Arg corresponds to the previous *Trp64Arg* variant) was found in 9 members. We describe herein the symptoms and GI involvement of these 9 affected members.

## Case presentation

### The proband

**Figure 1:**
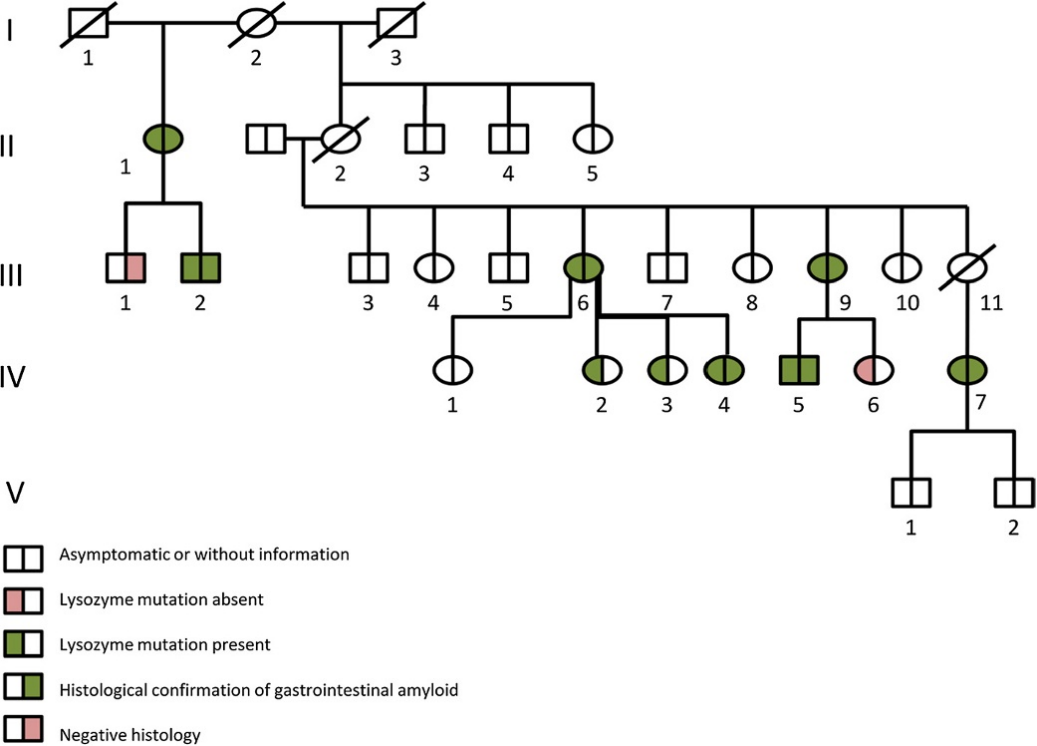
**Pedigree of the ALys family with gastrointestinal manifestation.** Subjects with the identification of the Alys mutation are shown with a left green half-square or half-circle. Individuals with white square or circle were not analyzed. Patients with a right green half-square or half-circle presented with gastrointestinal amyloidosis deposits on pathological analysis by red Congo staining.

The proband (III 6 in the family tree in the Figure 1) was a 51-year-old woman, from italian origin, with a longstanding history of gastric pain and symptoms of gastro esophageal reflux. She also complained of chronic cough. Previous upper endoscopies reported erythematous gastritis without helicobacter pylori infection and a bulbar ulcer. She was treated by proton pump inhibitors without success. One month later, control upper endoscopy showed persistent erythematous gastritis and biopsies revealed abnormal mucosal deposits characterized by positive Congo red stain with the pathognomonic birefringence under polarize light evoking amyloidosis. The patient also reported ocular and oral sicca syndrome and clinical examination revealed mild hepatomegaly. Neurological examination was normal. Serum protein electrophoresis and serum free light chains analysis excluded a monoclonal gammapathy and C reactive protein was normal. The patient had no proteins in the urine, urine blood casts and blood creatinin level were both normal.

On thoraco-abdominal CT-scan, only multiple inflammatory infracentimetric and centrimetric mesenteric lymph nodes with homogeneous hepatomegaly were noted. Echocardiography was normal. Genomic DNA analysis of the *LYZ* gene revealed a heterozygous single base-pair mutation (T to A substitution, TGG/AGG) in the codon 82 of exon 2 leading to the change of the amino acid from tryptophan to arginine (p.Trp82Arg in the new nomenclature, corresponds to the previously called *Trp64Arg* variant). After 4 years of follow up, the patient remained stable.

### Kindred

Clinical, biological, morphological and histological findings of the patient’s were then recorded.

Clinical, biological, morphological and histological findings of the patient’s were recorded prospectively or retrospectively. Gene analysis was part of standard care and was proposed to all the family’s member after obtaining their informed and written consent. This retrospective and descriptive study required no additional investigation to standard care and therefore did not require a specific statement of our ethical committee. All the patients were seen for physical examination in our clinical center and written consent was obtained for publication of the data.

### Histology and immunohistochemistry

Tissue biopsies, when available, were analyzed with the classical Congo red stain for amyloid deposits. Immunohistochemistry was only performed on the proband’s tissue biopsies using antibodies against amyloid A protein, kappa and anti lambda light chains and against lysozyme protein. Irrelevant monoclonal antibody and normal immunoglobulins were used as controls.

Tissues were obtained for 7 patients; five gastric biopsies, 4 colic biopsies and one gall-bladder, as showed in the family tree (Figure 1). All gastric biopsies and 3 of 4 colic biopsies showed amyloid deposits on red Congo stains. The gallbladder obtained after surgery for gallstones in patient IV4 showed also amyloid deposits in the gallbladder walls with the red Congo stain. Immunohistochemistry for amyloidosis typing was done only for the proband because of the unavailability of specific antibodies to Lyzozyme in all centers.

### DNA analysis

Genomic DNAs were extracted from peripheral blood leukocytes by a standard procedure after informed consent of the available family members. The five exons and flanking introns of the lysozyme gene were amplified by the polymerase chain reaction (PCR) using previously published primers and conditions [3]. Each PCR product was directly sequenced on both strands using a dye terminator cycle sequencing kit (Perkin Elmer, Norwalk, CT, USA) and the sequencing products were resolved on an automatic fluorescent DNA sequencer ABI Prism 377 (Applied Biosystems, Valencia, CA, USA). The nucleotide sequences of each exon were then compared with the published sequence of the lysozyme cDNA and the identified mutation was numbered according to the published cDNA sequence [4].

Nine subjects of the kindred (not including the proband) accepted after informed consent to be screened for the ALys mutation. Sequence analysis of lysozyme exon 2 revealed a heterozygous single base-pair transition from T to A of the first nucleotide position of codon 82 (TGG/AGG), in 9/10 tested members. This nucleotide substitution modifies the amino acid at position 82 in the mature protein from tryptophane to arginine. p.Trp82Arg in the new nomenclature, wich was previously called *Trp64Arg* variant).

### Kindred clinical presentation

Both parents of the proband were born in the Piedmont region in Italy (without known consanguinity). Her mother (II2) died aged of 52 years from meningeal hemorrhage and was not known to have gastrointestinal tract symptoms. The proband’s father was still in good health. The cause of death of her maternal grandmother (I2) and grandfather (I1) was unknown. The first symptoms, the clinical manifestations, the results of digestive investigations and follow-up of the 9 affected members of this family (including the proband) are shown in Table 1. All presented with GI symptoms. The most frequent symptom was heartburn related to endoscopic findings of non specific gastritis, ulcers (antral or bulbar) or oesophagitis. In all patients with endoscopic evaluation (7/9) typical amyloid deposits were shown on gastric or duodenal tissue biopsies with the red Congo staining. Interestingly, three members of this family had the diagnosis of inflammatory bowel disease: a proband’s sister (III9) who complained of rectal hemorrhage and chronic diarrhea. Lower endoscopy revealed left inflammatory erosive rectocolitis which led to the diagnosis of ulcerative colitis. She was treated with mesalazine. She also had chronic erythematous gastritis and colon polyps. In biopsies, amyloid deposits were observed in stomach and duonenum and in rectocolic biopsies. The second was a 27-year old woman (IV7) who presented with a history of abdominal pain and rectal hemorrhage with pancolitis leading to the diagnosis of ulcerative colitis. She improved under mesalazine. The third was a 24-year-old man who presented ileal ulcers on lower endoscopy suggesting the diagnosis of inflammatory bowel disease (IV5), biopsies only revealed amyloid deposits on ileal and colic biopsies without inflammatory lesions. Moreover, erythematous gastritis was noted on upper endoscopy with amyloid depositis in the antrum. Magnetic resonance imaging of ileon and fecal calprotectin were both normal. Three of the proband’s daughters were affected, all complained of gastro esophageal reflux symptoms. One of these suffered from gastric ulcer (IV4) and had surgery for gallstones at age 37. Typical amyloid deposits were found in her gall-bladder mucosa. The half-sister of the proband’s mother (II1) and his son (III2) were also affected. The 71-year-old aunt of the proband (II1) presented with a long history of gastro esophageal reflux symptoms and sicca syndrome. Iterative upper endoscopies noted peptic esophagitis, non hemorrhagic gastritis, antrum atrophy and duodenum diverticulitis. Amyloid depositis were found in the antrum and duodenal biopsies. Lower gastrointestinal tract endoscopy showed colon tubular polyposis and amyloid deposits. His son aged 41 (III2) presented only gastro esophageal reflux symptoms without gastritis on upper endoscopy. However, amyloid deposits were noted in the stomach. All the affected patients had normal echocardiography, normal kidney function, no inflammatory biological state, negative proteinuria and normal urine blood casts. None presented with neurological symptoms or abnormal neurological evaluation. All affected patients were alive with five years of follow up. Of note another sister (III4) and brother (III7) were known to have had chronic pancreatitis but they refused genetical testing. No further information could be obtained for II3, III5, III8 and III10.

**Table 1:** **Patients clinical and gastroenterological findings**

Patient (gender)	GI*symptoms	Age at diagnosis (years)	Upper GI tract findings ( ***pathological findings***)	Lower GI tract findings ( ***pathological findings***)	Treatment	Other clinical manifestations
II 1 (F)	Heartburn	71	Peptic esophagitis Esophagus Papilloma Non haemorrhagic gastritis Duodenum diverticulitis (*Duodenal and antrum amyloidosis plus antrum atrophy*)	Colon tubular polyp (*amyloidosis*)	PPI^#^	Sicca syndrome
III 2 (M)	Heartburn	41	(*Gastric amyloidosis*)	-	-	-
III 6 (F)	Heartburn Nausea Dyspepsia GOR**	51	Erythematous gastritis, Bulbar ulcer. (*Gastric amyloidosis*)	Normal	PPI^#^	Sicca syndrome Hepatomegaly Mesenteric lymphadenopathy
III 9 (F)	Rectal hemorrhage Diarrhea	46	Erythematous gastritis, Antrum ulcer (*Gastric and duodenal amyloidosis*)	Hemorrhagic rectocolitis Colon polyp (*recto colic amyloidosis* )	PPI^#^MSZ^§^	-
IV 2 (F)	Heartburn	28	-	-	-	-
IV 3 (F)	Heartburn	33	-	-	-	-
IV 4 (F)	Heartburn	34	Gastric ulcer	-	-	GallbladerAmyloidos
IV 5 (M)	Heartburn	24	Erythematous and atrophic gastritis (*Gastric amyloidosis*)	(*recto colic amyloidosis*)	-	-
IV 7 (F)	Abdominal pain Rectal hemorrhage	27	(*Gastric amyloidosis*)	IBD aspect (*recto colic amyloidosis* )	MSZ^§^	-

*Gastro intestinal (*GI*) ; **Gastroesophageal reflux (GOR); ^#^proton pump inhibitors (*PPI*); ^§^Mesalazine (*MSZ*).

## Conclusions

This new family of hereditary ALys associated with the p.Trp82Arg variant is of particular interest because the clinical manifestations were limited to the gastrointestinal tract with some affected individuals presenting with inflammatory bowel disease. Lysozyme is an hydrolytic enzyme mainly produced by liver, the digestive tract and by macrophages [5]. ALys deposits in affected patients have been found in gastrointestinal tract, liver, spleen, adrenal glands, muscles, vessels, lymph nodes, bone marrow and gallbladder [2–4, 6–9]. All affected individuals of this kindred complained of variable gastric pain and symptoms of gastro esophageal reflux. This confirms that ALys is very penetrant. The upper gastrointestinal tract endoscopic findings were unspecific in all affected patients showing erythematous gastritis with or without atrophy or gastric/bulbar ulcer, observed only in some patients. The symptoms were poorly improved by proton pump inhibitors. These upper gastrointestinal tract symptoms have already been reported in most of patients with ALys [4–11] and are probably related, as sicca syndrome, to the local production and deposit of the abnormal lysozyme protein. None of the patients of the kindred, even with longstanding symptoms, presented with digestive hemorrhage related to gastric ulcers. Only the systematic complementary analysis of the biopsies for amyloid deposits with the Congo red stain revealed the gastrointestinal tract amyloidosis. Because upper gastrointestinal tract manifestation can be relatively mild and non specific, as in this family, ALys is probably under diagnosed. This is illustrated by case II1 in whom the diagnosis, suggested by the discovery of ALys in the proband, was made at age 71 after numerous upper gastrointestinal tract endoscopies. Thus in cases of chronic gastritis or ulcers and a poor response to proton pump inhibitors, gastrointestinal tract amyloidosis should be evoked, and searched systematically on biopsies by red Congo staining. If positive and in case of a familial history patients should be screened for ALys. This can be done by immunohistochemistry with an anti lysozyme antibody and confirmed by DNA analysis.

Interestingly, three patients of the kindred were treated for inflammatory bowel disease and were found to have colonic ALys deposits. Two of them reported proctorrhagia. This suggests that ALys colonic amyloidosis, already reported in previous patients with ALys amyloidosis [5], could predispose to or mimic inflammatory bowel disease. This should be distinguished from rare reports of systemic AA amyloidosis secondary to inflammatory bowel disease [10].

In other type of amyloidosis, gastrointestinal tract involvement is very common but is often subclinical and the typical clinical presentations are also mostly nonspecific. They can present with macroglossia as observed in AL amyloidosis, hemorrhage, motility disorders, disturbance of bowel habit and malabsorption. Endoscopic and radiological features are also nonspecific, with the small intestine most commonly affected [12]. They can be distinguished by the analysis of the amyloid deposits on immunochemistry, the search of genetic variant associated with hereditary amyloidosis or pathologies associated with the acquired AA or AL amyloidosis.

Besides the gastrointestinal tract ALys can affect liver. Only the proband presented with mild hepatomegaly but the familial history noted no liver complications as hemorrhage or rupture as reported in previous families [11, 13, 14]. In this family ALys manifestations were very similar in affected individuals; there are heterogenous symptoms between different families of ALys but the clinical prentation seem to be homogenous in the same family.

Other manifestations of ALys were not presented by these family members except a sicca syndrome. It was present in the two most aged patients of this kindred as in 6/9 of previously reported patients with the W64R variant. ALys was not associated with clinical neurological or cardiac involvement [3, 4, 6–8, 15]. None of the patients of this family presented with renal insufficiency or proteinuria. Renal ALys amyloidosis was reported in patients with the D67H, the F57I and in some patient with the W64R lyzozyme variant. All the patients with the W64R variant and renal amyloidosis came from the family with the T to A substitution at codon 82. None of the patients from the three families with the T to C substitution at codon 82 (including this family) had renal involvement suggesting that other genetic factors are involved [15].

The families with the p.Trp82Arg variant already reported are either from French genetic background or from the Piedmont in Northern Italy. Two mutations at codon 82 of exon 2 lead to this variant: a T to A substitution (TGG/AGG) in three families of Italian origin and a T to C substitution (TGG/CGG) in one other unrelated family from French origin. The mother of the proband and her maternal grandparents were native from the Piedmont in Northern Italy as four previous families already reported [8]. Haplotype analysis has not been performed to date to link these three apparently unrelated families.

This new family of ALys, a form of hereditary non-neuropathic amyloidosis, confirms that gastrointestinal tract involvement is the most common manifestation of the disease. This new family suggests that renal involvement is uncommon in patient with the W64R variant. Symptoms are unspecific and mostly related to upper gastrointestinal tract involvement. For this reason we suggest that in case of atypical gastrointestinal tract disorders amyloidosis should be systematically searched by red Congo staining on standard biopsies. In case of amyloidosis and a familial history patients should be screened for ALys.

### Consents

Written informed consents forms were obtained for publication of this case report. Copies of the written consents are available for review by the Editor-in-Chief of this journal.
